# Social Isolation and Loneliness: Translating Current Research into Health Care Practices and Improving Awareness

**DOI:** 10.1093/geroni/igaa057.2512

**Published:** 2020-12-16

**Authors:** Colleen Galambos

**Affiliations:** University of Wisconsin Milwaukee Helen Bader School of Social Welfare, Milwaukee, Wisconsin, United States

## Abstract

This paper examines the evidence to support the need to translate current research into health care practices about social isolation and loneliness (SIL) among older adults. The health care system may be in the best position to identify those at highest risk—namely, older adults, whose only interactions are with members of the health care system. This paper reviews recommendations related to periodic assessments, including the use of validated tools to identify those at highest risk. Through this identification, clinicians and health care researchers may be able to use these findings to better target meaningful clinical and public health interventions. Additionally, a critical step toward preventing, mitigating, or eliminating negative health impacts will be to improve awareness about the problem and impact of SIL within the older adult population. This paper reviews recommendations for improving overall awareness by including SIL in national health strategies and public campaigns. Part of a symposium sponsored by Loneliness and Social Isolation Interest Group.

